# Supplementary material for: AT2 Receptor Mediated Activation of the Tyrosine Phosphatase PTP1B Blocks Caveolin-1 Enhanced Migration, Invasion and Metastasis of Cancer Cells
Source: Cancers (Basel). 2019 Sep 3;11(9):1299. doi: 10.3390/cancers11091299 (PMC6770525; doi:10.3390/cancers11091299)
Supplement: Supplementary file 1 [file cancers-11-01299-s001.pdf]

Supplementary Materials

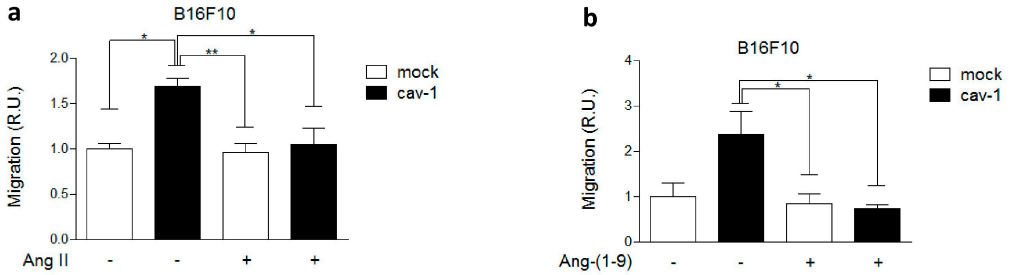

Figure S1. Ang II and Ang-(1-9) decrease migration of B16F10(cav-1) cells. Migration assays with B16F10 cells were performed as described in Materials and Methods. B16F10 cells were pretreated for 30 min with 1 μM Ang II or 3 h with 10 μM Ang-(1-9). Values obtained for migration were normalized to those of control cells without treatment. The graphs show values normalized to the average of the control condition (N = 3) (mean ±standard error of mean). Results obtained were compared statistically as described in Materials and Methods. Significant differences are indicated as ** $p \leq 0.01$, and * $p \leq 0.05$.

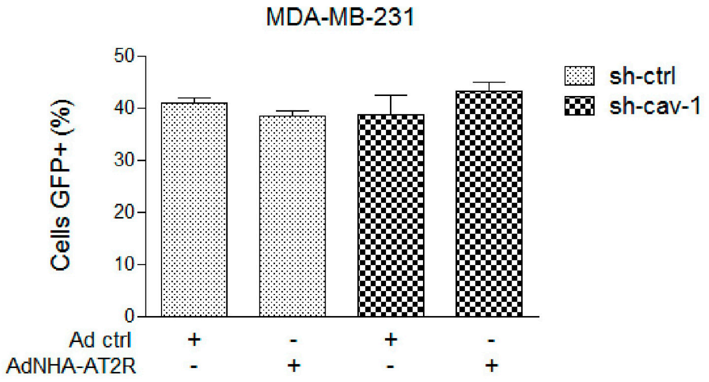

Figure S2. Green fluorescent protein **(**GFP)-positive B16F10 cells after transduction with adenovirus constructs**.** MDA-MB-231 cells were transduced with an adenovirus encoding AT2R which contained a bicistronic vector that permits co-expression of both the angiotensin receptor coupled to an HA-tag and the green fluorescent protein (GFP) (AdNHA-AT2R) or Adenovirus with a vector for GFP only was used as a control (Ad-ctrl). The cells were then trypsinized and counted. Subsequently, 50,000 were resuspended in 300 μL of phosphate-buffered saline (PBS) and supplemented with 2% fetal bovine serum. The analysis was performed by flow cytometry in a FACS Canto (Becton Dickinson, North Bend, WA, USA). The graphs show the percentage of GFP+ cells (N = 3).

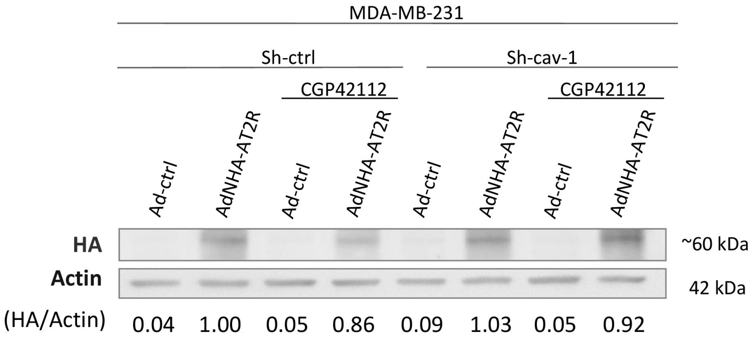

Figure S3. Expression analysis of the hemagglutinin (HA) tag in transduced MDA-MB-231. MDA-MB-231 cells transduced with AdNHA-AT2R or Ad-ctrl adenovirus and pretreated for 30 min with AT2R agonist N-α-Nicotinoyl-Tyr-Lys-(N-α-Z-Arg)-His-Pro-Ile (CGP42112) were analyzed by immunoblotting with antibodies against HA and β-actin (N = 1).

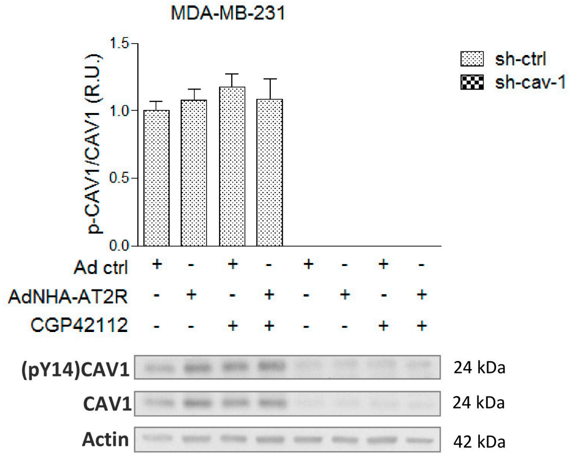

Figure S4. Tyrosine-14 phosphorylated caveolin-1 ((pY14)CAV1) levels in transduced MDA-MB-231 cells. (**a**) Extracts from MDA-MB-231 cells that were transduced with a adenovirus encoding AT2R which contained a bicistronic vector that permits co-expression of both the angiotensin receptor coupled to an HA-tag and the green fluorescent protein (GFP) AdNHA-AT2R or Adenovirus with a vector for GFP only was used as a control (Ad-ctrl) and pretreated for 30 min with AT2R agonist N-α-Nicotinoyl-Tyr-Lys-(N-α-Z-Arg)-His-Pro-Ile (CGP42112) (1 μM) were analyzed by western blotting using antibodies against pY14-CAV1, CAV1, and β-actin. The graph shows the pY14-CAV1/CAV1 ratios normalized to control conditions (N = 3).

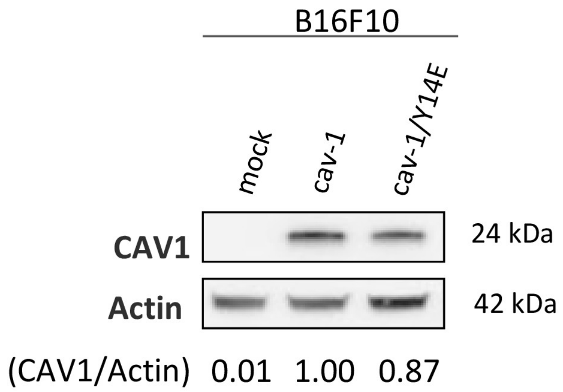

Figure S5. Analysis of caveolin-1 (CAV1) protein levels in B16F10(mock), B16F10(cav-1), and B16F10(cav-1/Y14E) cells**.** Extracts from B16F10(mock), B16F10(cav-1), and B16F10(cav-1/Y14E) were analyzed by immunoblotting with antibodies against CAV1 and β-actin. The numbers below individual lanes indicate the average CAV1/ β-actin ratio normalized to control conditions (N = 3).

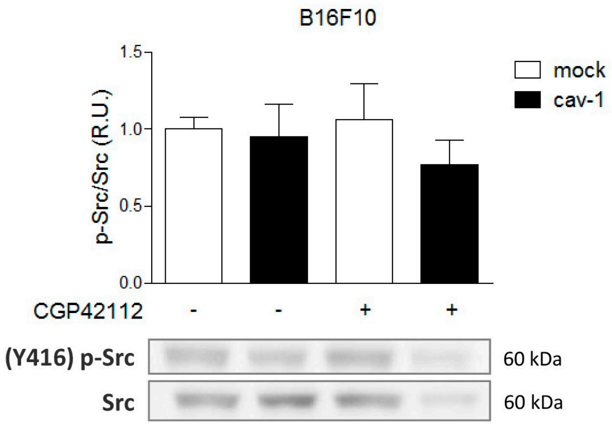

Figure S6. Tyrosine-416 phosphorylated Src kinase (Y416)p-Src levels in B16F10 cells. (**a**) Extracts from B16F10(cav-1) and B16F10(mock) cells that were pretreated for 30 min with CGP42112 (1 μM) were analyzed by Western blotting using antibodies against pY416-Src, Src, and β-actin. The graph shows the pY416-Src/Src ratios normalized to control conditions (N = 3).

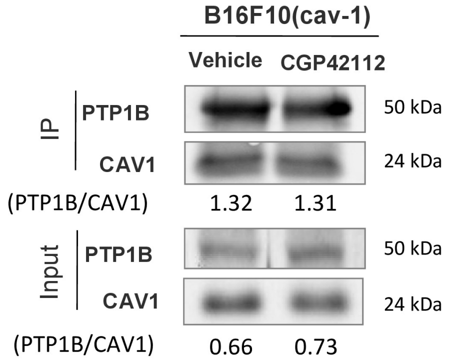

Figure S7. Immunoprecipitation of PTP1B. B16F10(cav-1) cells were treated for 30 min with either vehicle or AT2R agonist N-α-Nicotinoyl-Tyr-Lys-(N-α-Z-Arg)-His-Pro-Ile (CGP42112). Non-receptor protein tyrosine phosphatase 1B PTP1B was immunoprecipitated from cell lysates

(1.5 mg total protein) with a specific polyclonal antibody (2.5 µg per condition) for 3 h at 4 °C. The immunoprecipitates (IPs) and total cell lysates (input) were separated by sodium dodecyl sulfate polyacrylamide gel electrophoresis (SDS-PAGE), and PTP1B or CAV1 were detected using anti-PTP1B or anti-CAV1 antibodies, respectively (N = 1).

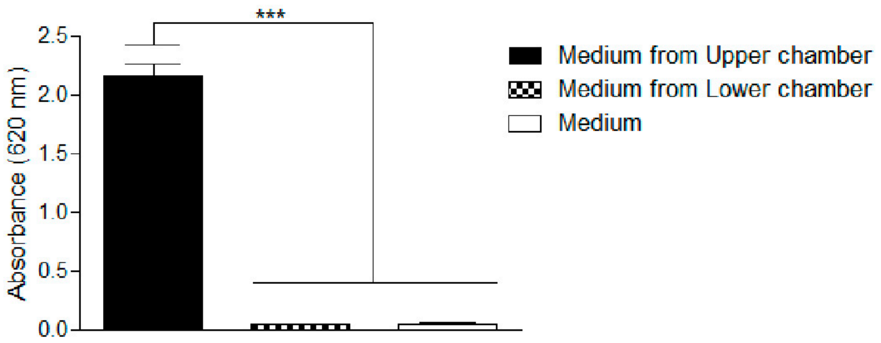

Figure S8. The EA.hy endothelial cell monolayers employed in Transendothelial migration assays (TEM) were impermeable to Dextran Blue. The permeability of endothelial cell monolayers was evaluated by testing the ability of such monolayers to retain Dextran Blue. For this, EA.hy cells (250,000) were seeded in the upper chamber of transwell inserts in complete medium and allowed to generate a monolayer of cells. After 72 h, the permeability of the monolayer was evaluated by adding complete medium with Dextran Blue (10 mM) to the upper chamber and medium only to the lower chamber. Then, after 30 min, the absorbance was determined at 620 nm. The absence of changes in the lower chamber were taken to be indicative of a sealed cell monolayer. Significant differences are indicated (N = 3). *** $p \leq 0.001$.
